# Stimulus Contrast and Retinogeniculate Signal Processing

**DOI:** 10.3389/fncir.2016.00008

**Published:** 2016-02-19

**Authors:** Daniel L. Rathbun, Henry J. Alitto, David K. Warland, W. Martin Usrey

**Affiliations:** ^1^Center for Neuroscience, University of CaliforniaDavis, Davis, CA, USA; ^2^Institute for Ophthalmology and Center for Integrative Neuroscience, University of TübingenTübingen, Germany; ^3^Department of Neurobiology, Physiology, and Behavior, University of CaliforniaDavis, Davis, CA, USA

**Keywords:** retina, LGN, coding, vision, thalamus

## Abstract

Neuronal signals conveying luminance contrast play a key role in nearly all aspects of perception, including depth perception, texture discrimination, and motion perception. Although much is known about the retinal mechanisms responsible for encoding contrast information, relatively little is known about the relationship between stimulus contrast and the processing of neuronal signals between visual structures. Here, we describe simultaneous recordings from monosynaptically connected retinal ganglion cells and lateral geniculate nucleus (LGN) neurons in the cat to determine how stimulus contrast affects the communication of visual signals between the two structures. Our results indicate that: (1) LGN neurons typically reach their half-maximal response at lower contrasts than their individual retinal inputs and (2) LGN neurons exhibit greater contrast-dependent phase advance (CDPA) than their retinal inputs. Further analyses suggests that increased sensitivity relies on spatial convergence of multiple retinal inputs, while increased CDPA is achieved, in part, on temporal summation of arriving signals.

## Introduction

All visual information leaving the eye is conveyed in the spiking activity of retinal ganglion cells. Given the limited dynamic range of these cells and the dramatically varying statistics of visual stimuli in the natural world, efficient encoding of visual information requires processing that responds to the statistics of the visual input. Contrast gain control is a prominent mechanism used by the visual system to meet the challenge of encoding visual information in diverse visual environments. Contrast gain control refers to the nonlinear receptive field property whereby a neuron’s gain and temporal dynamics are dependent upon stimulus contrast. Specifically, the response gain of neurons in the early visual system, including the retina, LGN, and V1 decreases as stimulus contrast increases, causing contrast response functions to saturate at contrasts below 100%. Additionally, as stimulus contrast increases, these same visual neurons become more responsive to stimuli with high temporal frequencies, and they exhibit a contrast-dependent phase advance (CDPA) in their responses to periodic stimuli. Although contrast gain control is first established within the retina, an open and unresolved question is how contrast gain control is enhanced between the retina and the LGN.

The axons of retinal ganglion cells target several central structures, including the lateral geniculate nucleus (LGN) of the thalamus which in turn provides monosynaptic excitation to primary visual cortex (V1). Although the response properties of retinal ganglion cells and LGN neurons are generally quite similar (Hubel and Wiesel, 1961; Cleland et al., 1971; Levick et al., 1972; So and Shapley, 1981; Lee et al., 1983; Cleland and Lee, 1985; Kaplan et al., 1987; Mastronarde, 1987, 1992; Usrey et al., 1999; Rathbun et al., 2010), there are significant differences, and one of the most prominent of these involves the relationship between stimulus contrast and neuronal activity. In particular, LGN neurons display greater contrast gain control than their retinal inputs (Kaplan et al., 1987; Scholl et al., 2012; but see Sclar, 1987).

Factors that influence the feedforward communication of retinal signals to LGN neurons include the convergence of retinal inputs onto individual LGN neurons and the temporal summation of arriving signals. Studies in the cat indicate that LGN neurons typically receive convergent input from 2 to 5 retinal ganglion cells (Cleland et al., 1971; Cleland, 1986; Hamos et al., 1987; Mastronarde, 1992; Usrey et al., 1999; Reid and Usrey, 2004; Martinez et al., 2014). Likewise, the excitatory postsynaptic potentials (EPSPs) evoked from the spikes of individual retinal axons interact over interspike intervals (ISIs) of up to ~30 ms to increase the likelihood of bringing an LGN neuron to spike threshold (Mastronarde, 1987; Usrey et al., 1998; Levine and Cleland, 2001; Rowe and Fischer, 2001; Carandini et al., 2007; Sincich et al., 2007; Weyand, 2007; Rathbun et al., 2010). The goal of this study was to determine whether and how convergence and temporal summation contribute to the transmission and processing of contrast information between the retina and LGN.

To determine the influence of stimulus contrast on retinogeniculate communication and visual processing in the retina and LGN, we made simultaneous recordings from monosynaptically connected retinal ganglion cells and LGN neurons in the anesthetized cat and measured neuronal responses to drifting sinusoidal gratings that varied in stimulus contrast. Consistent with predictions from past studies (Kaplan et al., 1987; Scholl et al., 2012), our results demonstrate that the contrast needed to evoke a half-maximal response (*C_50_*) is lower for LGN neurons than for their individual retinal afferents. Further analysis suggests that this effect relies on the integration of multiple retinal inputs by individual LGN neurons. Our results also reveal that CDPA—a hallmark of contrast gain control—is significantly greater for LGN neurons than for their individual retinal afferents. To probe the underlying mechanism responsible for the CDPA changes, we applied a model of ISI-based filtering to recorded retinal spike trains. Results from this effort reveal that an ISI-based filtering mechanism of retinal spikes can produce CDPA in target neurons. Taken together, these results indicate that the LGN is more than a simple relay station, as it adjusts both the sensitivity and timing of visual signals en route from retina to cortex.

## Materials and Methods

### Animal Preparation

Six adult cats of both sexes were used in this study. All surgical and experimental procedures conformed to NIH guidelines and were carried out with the approval of the Animal Care and Use Committee at the University of California, Davis. Anesthesia was induced with ketamine (10 mg/kg, IM) and thiopental sodium (10 mg/kg, IV; supplemented as needed). Animals received a tracheotomy and were placed in a stereotaxic apparatus where the temperature, electrocardiogram (ECG), electroencephalogram (EEG), and expired CO_2_ were monitored continuously for the duration of the experiment. All wound margins were infused with lidocaine and anesthesia was maintained with a continuous infusion of thiopental sodium (2–3 mg/kg/h, IV). If physiological monitoring indicated a low level of anesthesia, additional thiopental was given and the rate of continuous infusion was increased. A midline scalp incision was made and the cortical surface above the LGN was exposed through a craniotomy which was filled with agarose. The lateral margin of each eye was dissected and each sclera was glued to a rigid post mounted on the stereotaxic frame. These posts secured the eyes and facilitated the introduction of a trans-scleral guide tube for retinal recordings. The nictitating membranes were retracted with 10% phenylephrine and flurbiprofen sodium drops were administered (1.5 mg/h) to prevent miosis. The eyes were refracted, fitted with appropriate contact lenses, and focused on a tangent screen located 172 cm in front of the animal. The positions of area centralis and the optic disk were plotted by back-projecting the retinal vasculature of each eye onto the tangent screen. Once all surgical procedures were complete, animals were paralyzed with vecuronium bromide (0.2 mg/kg/h, IV) and mechanically respired.

### Electrophysiological Recording and Visual Stimuli

A multielectrode array (Thomas Recording, Marburg, Germany) was used to record from individual LGN neurons from seven independently positioned microelectrodes. The locations of receptive fields measured using the array were used to guide the placement of the retinal electrode. To record from retinal ganglion cells, a tungsten-in-glass microelectrode was introduced into the posterior chamber of the eye through a guide tube and positioned using a custom-made manipulator. Neural responses were amplified, filtered and recorded to a personal computer with a Power 1401 data acquisition interface and the Spike 2 software package (Cambridge Electronic Design, Cambridge, UK). The spikes from individual neurons were isolated using template matching and parametric clustering.

Visual stimuli were created with a VSG 2/5 visual stimulus generator (Cambridge Research Systems, Rochester, UK) and presented on a gamma-calibrated Sony monitor with a mean luminance of 38 cd/m^2^. Receptive fields were mapped in space and time using a binary white-noise stimulus and reverse-correlation analysis (Reid et al., 1997; Rathbun et al., 2007). To examine the influence of stimulus contrast on the timing and strength of neuronal responses and the efficacy of retinogeniculate communication, recordings were made while neurons were excited with drifting sinusoidal gratings (4 Hz, optimal spatial frequency). Gratings were shown for 4 s, followed by 4 s of mean gray, at 10 different contrast levels (random order), spaced logarithmically from 1 to 100%. The complete stimulus set was presented 100–300 times, as permitted by recording stability.

X and Y cells were distinguished on the basis of receptive field size, response latency, and time course of response (Usrey et al., 1999). Although recordings were made from both X and Y cells in the LGN, there was a heavy sampling bias for Y-type cells in the retina (see also Rathbun et al., 2010). Consequently, only Y-cell pairs were examined in this study. It is worth noting that Y-type cells are well suited for studying contrast-dependent processing, as they generally exhibit stronger contrast gain control and CDPA than X cells (Shapley and Victor, 1978).

### Data Analysis

#### Cross Correlation Analysis

**Figure 1:**
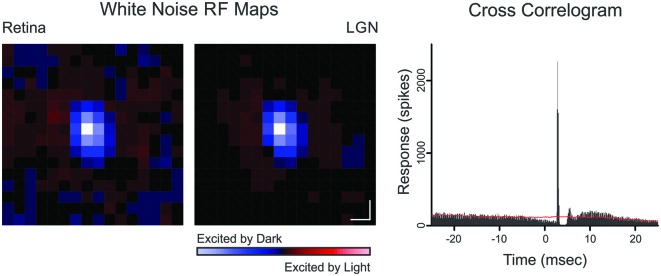
**Spatial receptive field maps and cross-correlogram for a retinogeniculate OFF-cell pair (Pair 10).** Receptive field maps are the spike triggered average of a white-noise stimulus (as described in Rathbun et al., 2010). Each map was normalized to the pixel of highest magnitude where red indicates ON responses and blue indicates OFF responses. Scale bar denotes 1° of visual angle. The cross-correlogram was calculated from a 6000 s recording that contained 154,152 retinal spikes and 21,697 LGN spikes. *Red line* indicates shuffle-corrected baseline.

Cross-correlation analysis was used to identify monosynaptically connected retinogeniculate cell pairs (Figure 1). A cross-correlogram was generated by creating a histogram of LGN spikes relative to each retinal spike. The presence of a sharp, short-latency peak in the cross-correlogram was taken as evidence of a monosynaptically-connected pair of cells (Cleland et al., 1971). For quantitative analysis, cross-correlation bins contributing to the peak were identified using a bin size of 0.1 ms. The peak bin was first identified and all neighboring bins greater than three standard deviations above the baseline mean were considered part of the peak; where the baseline consisted of bins ranging from 30 to 50 ms on either side of the peak bin. Because each count in the cross-correlogram peak represents a single retinal spike that was relayed by the LGN neuron to cortex, these retinal spikes were termed “relayed” spikes whereas the remaining retinal spikes were termed “non-relayed” spikes. Likewise, LGN spikes that contributed to the cross-correlogram peak were termed “triggered” spikes, indicating that they were evoked by the simultaneously recorded retinal ganglion cell; and the remaining LGN spikes were termed “non-triggered” spikes (i.e., spontaneous spikes or spikes evoked from a source other than the simultaneously recorded retinal ganglion cell). Two values used to quantify the strength of communication between a simultaneously recorded retinal ganglion cell and LGN neuron are efficacy and contribution (Levick et al., 1972), where efficacy is the percentage of retinal spikes that evoked an LGN spike (i.e., relayed spikes) and contribution is the percentage of LGN spikes evoked from a particular retinal input (i.e., triggered spikes).

#### Response Curve Fitting

To determine the amplitude and phase of responses to drifting gratings, spike times were expressed relative to the phase of the sinusoid cycle, producing a cyclic histogram for each contrast. A constrained nonlinear optimization procedure (MATLAB function: *nlinfit*; The Mathworks, Natick, MA, USA) was used to fit each cyclic histogram with the positive-only rectification of the following sinusoidal equation:

(1) R(t)=A*sin (ω*t+θ)+b

where *R(t)* is the magnitude of the cyclic histogram at time *t*, *A* is the response amplitude at the modulation frequency, *ω* is angular frequency of the drifting stimulus in radians per second, *t* is time in seconds, *θ* is the response phase determined by the vector sum of phases for all spikes in the cyclic histogram, and the baseline (*b*) indicates the value below which the sinusoid is rectified. The baseline was constrained to range between –*A* and 2 * *A*. For each spike train, cyclic histograms were fitted sequentially from low to high contrast with the additional constraint that *b* was monotonically decreasing. This procedure was found to produce more useful estimates of the modulated response amplitude and response phase, independent of contrast-induced changes in rectification, than a standard Fourier decomposition algorithm available in MATLAB (*fft*), and is analogous to estimation of the F1.

For each spike train, the contrast response function was fitted with a hyperbolic ratio (Albrecht and Hamilton, 1982; MATLAB function: *fminunc*; The Mathworks, Natick, MA, USA):

(2) $$R(C)=\frac{R_{\max}*C^{n}}{C^{n}+C_{50}^{n}}+b$$

where *R(C)* is the response for a given contrast *C*, *R_max_* is the maximal response amplitude across contrasts, the exponential *n* reflects the sensitivity of the response function, *C_50_* is the contrast corresponding to 50% of the maximal response, and *b* denotes baseline and was set as the response to the lowest contrast (1%).

As contrast increased, response phase was often observed to advance progressively earlier in the stimulus cycle. This phenomenon will be referred to as CDPA to distinguish it from absolute phase advance relative to the stimulus. In order to quantify CDPA, a first-order polynomial was fit to the curve of phase vs. log (contrast) over the middle six contrasts presented (range: 2.78–35.94% contrast; see Figure 4). The resultant slope quantifies CDPA magnitude in units of degree/octave. In earlier reports, CDPA has sometimes been expressed as the amount of phase advance over the eightfold range from 1.25 to 10% contrast (Shapley and Victor, 1978; Sclar, 1987). Because, we found that phase estimates were often unreliable at very low levels of contrast, we chose to exclude response values from contrasts less than 2.78%. The upper contrast limit was chosen to exclude saturation effects and falls near or below the *C_50_* for all curves.

#### Modeling Contrast-Dependent Phase Advance

Results from previous studies show that retinal spikes following short ISIs are more effective in evoking LGN responses than retinal spikes following longer ISIs (Mastronarde, 1987; Usrey et al., 1998; Levine and Cleland, 2001; Rowe and Fischer, 2001; Carandini et al., 2007; Sincich et al., 2007; Weyand, 2007; Rathbun et al., 2010). Given that the mean firing rate of retinal ganglion cells typically increases as contrast increases, there will necessarily be a shift in the distribution of ISIs as a function of contrast. To determine the extent to which the augmentation of CDPA measured in the LGN relative to the retina could be accounted for by the contrast-dependent shift in ISI distribution, we generated simulated LGN spike trains based on weighting actual retinal spikes in experimentally recorded data according to the ISI vs. spike efficacy relationship curve calculated from responses to white-noise stimulation (Rathbun et al., 2010). For example, if 30% of retinal spikes following an ISI of 10–15 ms were found to evoke an LGN spike compared to only 15% of retinal spikes following an ISI of 15–20 ms, then these two groups of retinal spikes were assigned weights of 0.3 and 0.15, respectively (Alitto and Usrey, 2015) as their contributions to the simulated LGN spike train. This process was repeated for every retinal spike and simulated spike trains were analyzed in exactly the same manner as those from real LGN neurons, as described above.

#### Statistical Analysis

Unless otherwise indicated, population data is summarized by the mean and standard error of the mean. Wilcoxon’s signed-rank test (MATLAB function: *signrank*; The Mathworks, Natick, MA, USA) was used to determine p values for all pair-wise statistical tests.

## Results

### Comparing Contrast Response Functions in Retina and LGN

**Figure 2:**
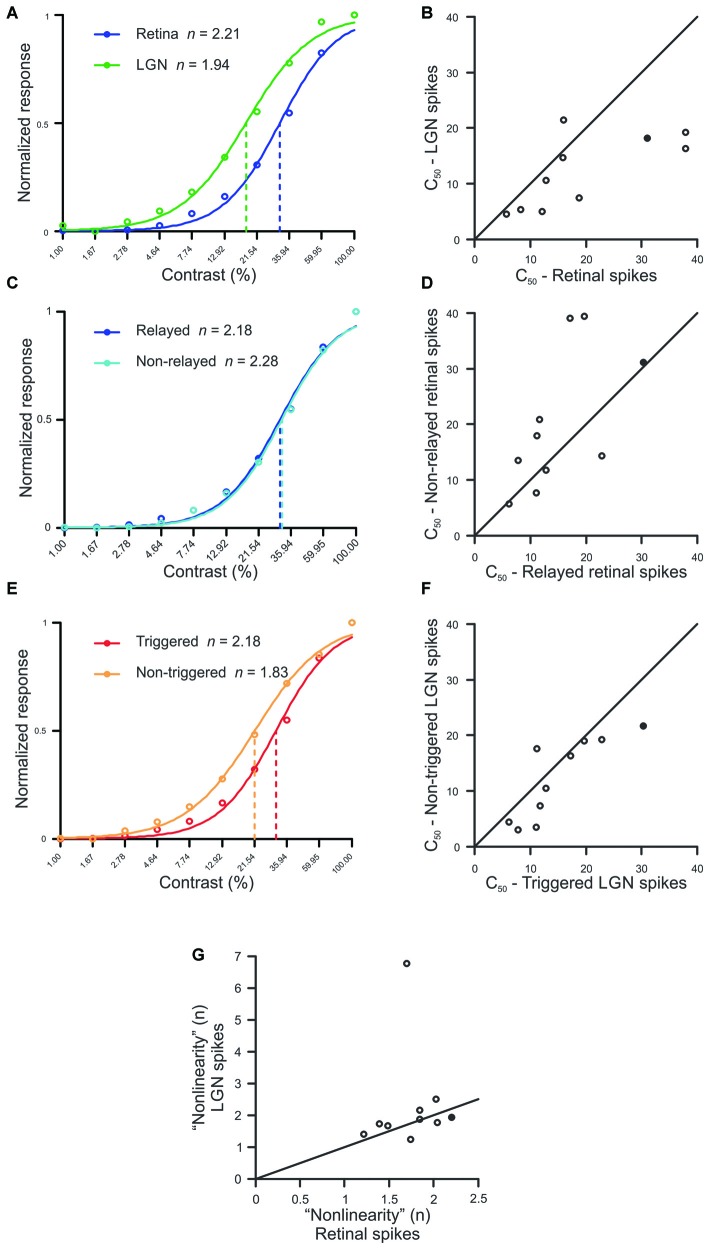
**Comparison between spike classes for hyperbolic ratio fit parameters. (A,C,E)** Contrast response functions for a single example pair (Pair 16, filled symbol in **B,D,F,G**) where raw data is plotted with *circles*, *solid lines* denote the hyperbolic ratio fit, and *dashed lines* indicate the *C_50_*. **(B,D,F)** Scatterplots of *C_50_* for retinal and LGN spikes **(B)**, relayed and non-relayed retinal spikes **(D)**, and triggered and non-triggered LGN spikes **(F)**. **(G)** Scatterplot comparing the exponent *n* between retinal and LGN spike trains. In all scatterplots, solid diagonal line denotes unity.

Simultaneous recordings were made from 10 pairs of monosynaptically-connected retinal ganglion cells and LGN neurons in order to study the influence of stimulus contrast on neuronal responses across the retinogeniculate synapse (see “Materials and Methods” Section; Figure 1). Responses to contrast-variant drifting grating stimuli (4 Hz, optimal spatial frequency) were used to determine the *C_50_*, defined as the contrast to evoke 50% of maximum response, for each neuron in our sample (*n* = 19; all Y cells). The *C_50_* is therefore a good metric for contrast gain control as it tends to be lowest for neurons that exhibit greater contrast saturation. Across our sample of connected cells, LGN neurons typically had significantly lower *C_50_* values than their simultaneously recorded retinal input (Figures 2A,B; *p* = 0.02), indicating that LGN neurons display greater contrast gain control than their retinal counterparts.

To determine whether the decrease in *C_50_* that occurred between pre-and postsynaptic neurons was the result of a selective filtering of retinal spikes, we compared *C_50_* values for relayed and non-relayed retinal spikes (see “Materials and Methods” Section). As shown in Figures 2C,D, there was not a significant difference in *C_50_* between the two classes of retinal spikes (*p* = 0.23). Thus, it seems unlikely that the difference in *C_50_* values between retina and LGN can be attributed to the selective filtering of spikes generated by the simultaneously recorded retinal ganglion cells.

Estimates indicate that individual LGN neurons in the cat typically receive monosynaptic input from approximately 2–5 retinal ganglion cells (Cleland et al., 1971; Cleland, 1986; Hamos et al., 1987; Mastronarde, 1992; Usrey et al., 1999; Reid and Usrey, 2004; Martinez et al., 2014). To address the possibility that this convergence contributes to the shift in *C_50_* between retinal ganglion cells and LGN neurons, we divided the spikes generated by each LGN neuron in our sample into two categories: those evoked (or “triggered”) by the simultaneously recorded retinal ganglion cell, and those evoked from other sources (“non-triggered”), including other retinal ganglion cells. Across our sample of cells, *C_50_* values were significantly lower for non-triggered LGN spikes compared to triggered spikes (Figures 2E,F; *p* < 0.05). This finding is consistent with the idea that an LGN cell’s contrast response function is shifted in the direction of its most sensitive input which, because of convergence, is likely to be an input other than the simultaneously recorded retinal ganglion cell.

We next examined whether the exponent “*n*” from the equation used to fit the contrast response functions differed between connected retinal and LGN neurons (see “Materials and Methods” Section). In general, this exponent can be taken to quantify the linearity of the contrast response function, with small exponents indicating a relatively linear curve, and larger exponents indicating nonlinear expansion below the inflection point and compression above it. Consistent with Duong and Freeman (2008), we found that the expansive nonlinearity at low contrasts (*n* > 1) was present for all of the Y-type retinal ganglion cells and LGN neurons in our sample. However, we did not find a significant difference in the exponent term *n* between pre- and postsynaptic neurons, suggesting that this feature of the contrast response function is passed on, unaltered, from retina to LGN (Figure 2G; *p* = 0.375). It is worth noting that the single outlier in Figure 2G corresponds to an LGN neuron which exhibited a significantly stronger F2/F1 ratio than any other cell in this study. This pair of cells also represents the only pair in the sample in which the classification of the retinal ganglion cell was not clear (the LGN neuron was Y type). With that said, this cell pair did not differ from the rest of the sample in all of the other analyses performed.

### Comparing Contrast-Dependent Phase Advance in Retina and LGN

**Figure 3:**
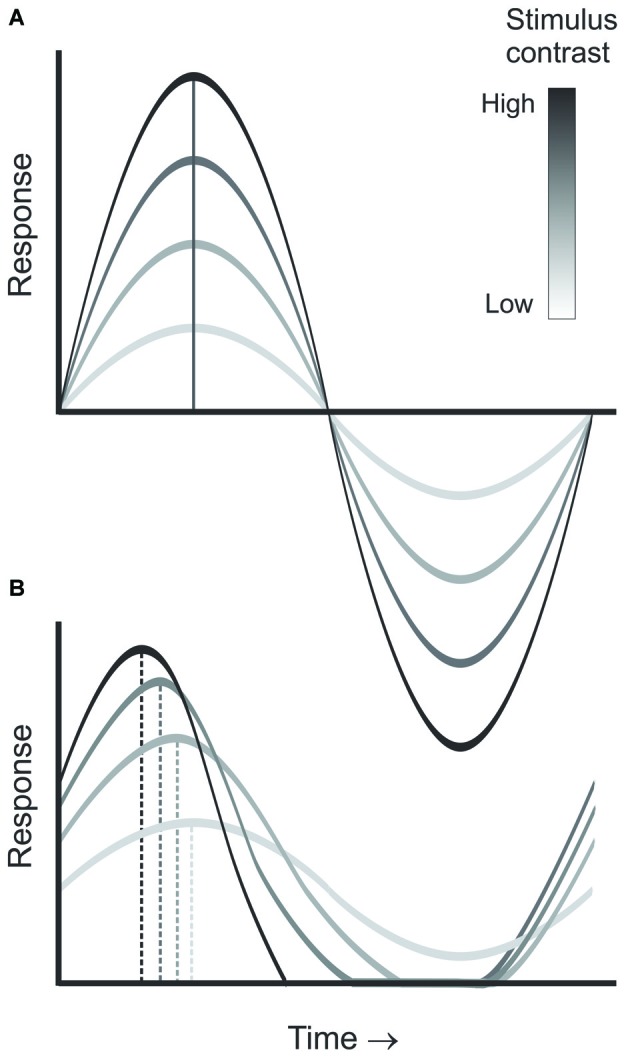
**Diagram demonstrating contrast dependent phase advance.** As stimulus contrast increases **(A)**, the neuron’s temporal integration window becomes shorter, leading to increased transience as illustrated by the responses at each contrast **(B)**, thus, leading to a progressive advance in the response phase relative to the stimulus cycle with increasing contrast.

CDPA is another hallmark of nonlinear processing in the early visual system. As contrast increases, neurons in the retina, LGN and visual cortex are reported to respond progressively earlier relative to the phase of the stimulus (Shapley and Victor, 1978; Dean and Tolhurst, 1986; Sclar, 1987; Alitto and Usrey, 2004). While this effect is partly due to a decrease in latency with increasing contrast, it is also believed to result from an increase in transience that is induced by contrast gain control mechanisms (Figure 3, Shapley and Victor, 1978). Importantly, models of geniculocortical processing often incorporate an increase in the CDPA from LGN to cortex (Kayser et al., 2001). While a similar increase from retina to LGN has been hypothesized, this increase has yet to be demonstrated directly. One study that examined this question through a meta-analysis of the existing literature found no difference between the two structures (Kayser et al., 2001). Paired-cell recording, however, provide a particularly sensitive tool to examine directly subtle differences in the responses of LGN neurons and their retinal inputs.

**Figure 4:**
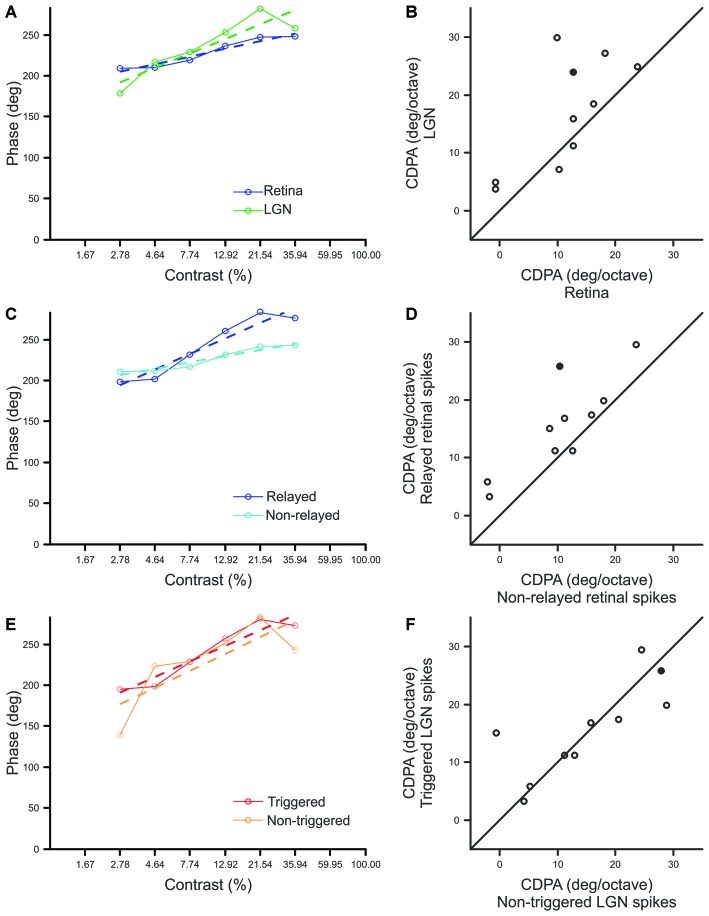
**Comparison of contrast-dependent phase advance (CDPA) between spike classes. (A,C,E)** Response phase at each contrast for a single example pair (Pair 15, filled symbol in **B,D,F**) where *dashed lines* indicate best fit line to the data, *solid lines*, over the range of 2.78–35.94% contrast. The phase of responses to 1% contrast are omitted to improve graph legibility. CDPA is given by the slope of the best fit line in degree/octave. **(B,D,F)** Scatterplots of CDPA for retinal and LGN spikes **(B)**, relayed and non-relayed retinal spikes **(D)**, and triggered and non-triggered LGN spikes **(F)**.

Consistent with previous reports, our sample of Y-type retinal ganglion cells exhibited an average phase advance of ~55° over the 2.78–35.94% contrast ranges (Shapley and Victor, 1978 reported ~50°). More importantly, a comparison of CDPA between simultaneously recorded retinal ganglion cells and LGN neurons revealed that CDPA is significantly greater for LGN neurons than their recorded retinal inputs (*p* = 0.027; Figures 4A,B). This finding confirms the hypothesis that the influence of contrast gain control progressively increases throughout the early visual system. In examining differences between spike classes, we found that relayed retinal spikes exhibited significantly greater CDPA values than non-relayed retinal spikes (Figures 4C,D; *p* = 0.004), suggesting that the increase in CDPA from retina to LGN relies, in part, on a selective filtering of retinal spikes. Across our sample of cell pairs, there was not a significant difference between CDPA values calculated from triggered and non-triggered LGN spikes (Figures 4E,F; *p* = 0.777), suggesting that signals from simultaneously recorded and non-recorded retinal ganglion cells experienced comparable levels of CDPA.

### Modeling Changes in Contrast-Dependent Phase Advance

**Figure 5:**
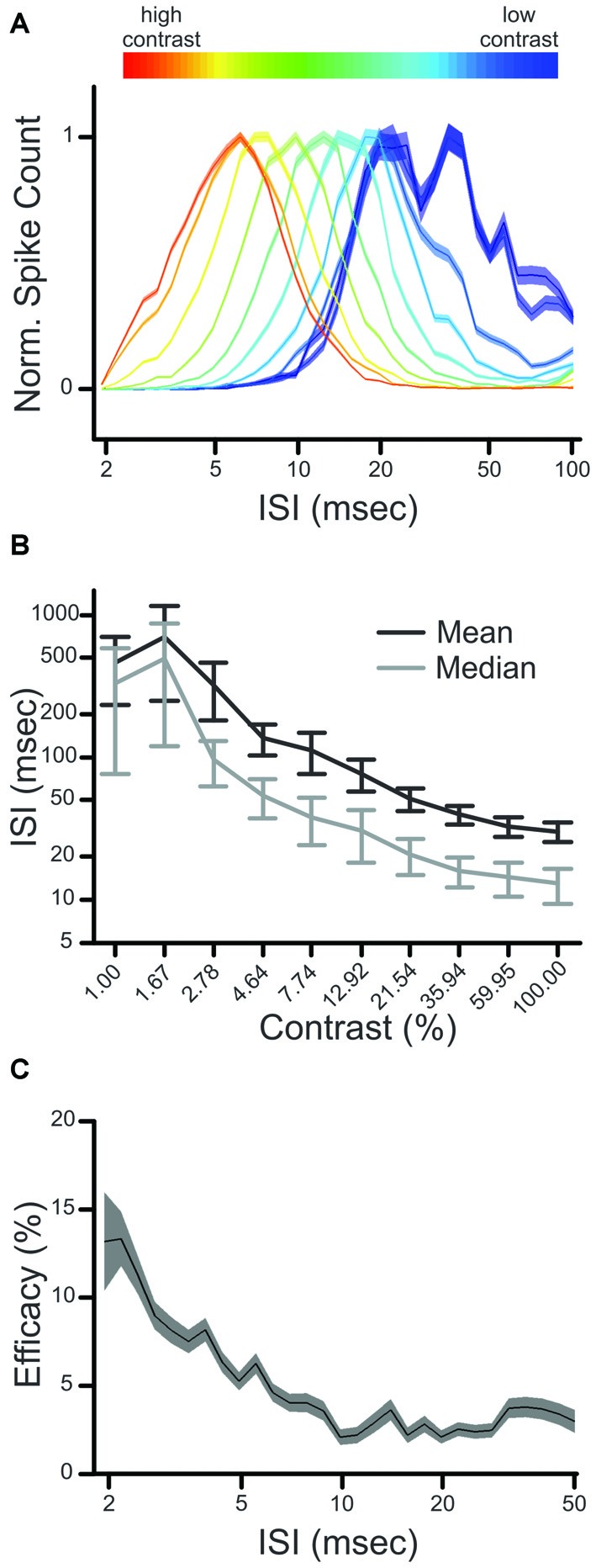
**Influence of contrast on interspike interval (ISI) distribution and ISI-based filtering. (A)** Normalized ISI distributions of a single retinal neuron (Pair 2). The influence of contrast on ISI distribution is indicated by color (cold to hot), where the ISI distribution produced by the lowest contrast is plotted in *blue*, and that of the highest contrast is in *red*. **(B)** influence of contrast on mean and median ISI for all Y-class retinal ganglion cells. **(C)** ISI-based efficacy filter for the sample pair. Shaded regions in **(A,C)** denote error bars.

Previous research has shown that the retinal interspike interval (ISI) has a strong influence on the generation of postsynaptic responses in the LGN, as retinal spikes following short ISIs (<30 ms) are significantly more likely to evoke a postsynaptic spike than retinal spikes following longer ISIs (Carandini et al., 2007). Given this, we asked whether an ISI-based filter for retinogeniculate communication could underlie the increase in CDPA, described above. As a first step, we determined the influence of stimulus contrast on the distribution of ISIs in retinal spike trains. Across our sample of retinal ganglion cells, the distribution of ISIs was shifted toward lower ISIs as contrast increased (Figures 5A,B), consistent with the expected inverse relationship between firing rate and ISI.

**Figure 6:**
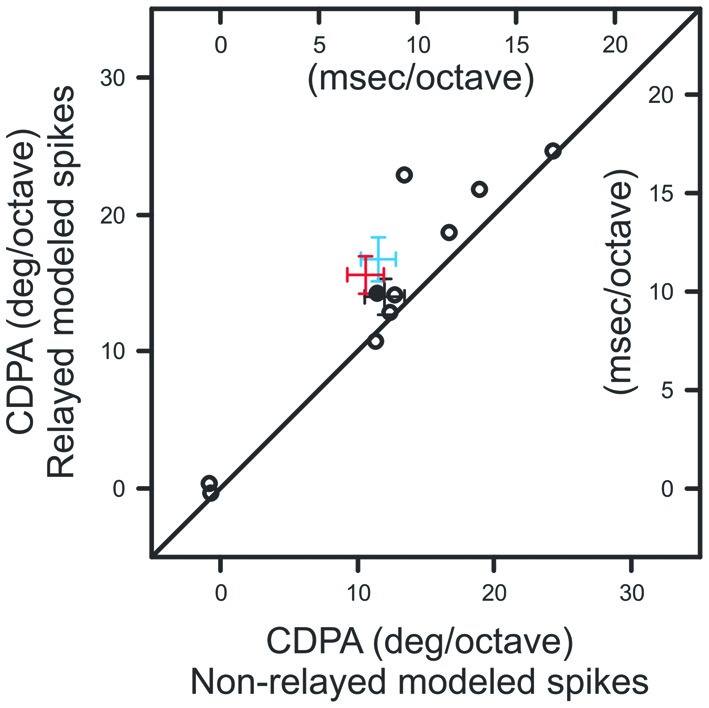
**Scatterplot showing CDPA of modeled relayed and non-relayed spikes.** *Black diagonal* denotes unity. Population means and SEM from Figures 4B,D are indicated in *blue* and *red*, respectively.

We next quantified the relationship between ISI and the efficacy of retinal spikes in evoking postsynaptic responses for each retinogeniculate pair of simultaneously recorded neurons in our sample. Similar to previous results (Rathbun et al., 2010), retinal spikes with the shortest preceding ISIs were most effective in evoking postsynaptic responses (Figure 5C). Finally, we combined the ISI distributions that were determined for each contrast with the ISI filter to determine the extent to which the filter could reproduce the CDPA effect (see “Materials and Methods” Section). This model tests the hypothesis that simple, temporal filtering of retinal spikes, as estimated by the ISI-spike efficacy curve, can directly contribute to the increased CDPA of LGN neurons. For the modeled data, as with the original data, we found that relayed spikes had significantly greater CDPA when compared to non-relayed spikes (Figure 6; *p* = 0.014). While the magnitude of this effect was less than what was found in the original data (2.0 ± 0.1 vs. 5.0 ± 1.6 degree/octave, respectively; see “Discussion” Section), this result suggests that temporal filtering indeed plays a significant role in the increased CDPA that occurs across the retinogeniculate synapse.

## Discussion

Stimulus contrast is one of the most salient features encoded in the activity of neurons in the retina and LGN. Indeed, the center/surround organization of retinal and LGN receptive fields is ideal for detecting local changes in contrast. Although the spatial organization of retinal and LGN receptive fields are quite similar, past studies indicate that LGN neurons display greater contrast gain control, on average, than retinal ganglion cells. Given the significance of stimulus contrast for nearly all aspects of visual processing, it is surprising that relatively little attention has been paid to how contrast responses are transformed as they pass from retina to LGN (Kaplan et al., 1987; Cheng et al., 1995).

Here, we compared the responses of monosynaptically connected Y-type retinal ganglion cells and LGN neurons in the cat as a function of stimulus contrast. Consistent with the view that nearly all spikes in the LGN are triggered by retinal action potentials (Kaplan and Shapley, 1984; Sincich et al., 2007), we found the overall shape of contrast response functions to be similar for simultaneously recorded retinal ganglion cells and LGN neurons. However, our results also demonstrate that the semisaturation contrast (*C_50_*) is significantly lower for LGN neurons compared to their simultaneously recorded retinal input, indicating that contrast gain control is enhanced by the LGN and that LGN responses begin to saturate at lower contrasts than their retinal inputs. Further analysis suggests that this decrease in *C_50_* relies, at least in part, on convergence of multiple retinal inputs onto individual LGN neurons. With convergence, LGN neurons receive input from an ensemble of retinal ganglion cells that have a range of sensitivity profiles, and the most sensitive of these inputs will be those that influence LGN activity under low contrast conditions, thereby shifting *C_50_* values toward lower contrasts. Because individual LGN neurons are estimated to receive convergent input from approximately 2–5 retinal ganglion cells, our recording configuration was unlikely to include the most sensitive retinal input, a view consistent with the finding that non-triggered retinal spikes (spikes evoked from other sources) have lower *C_50_* values than spikes triggered by the simultaneously recorded retinal ganglion cell.

Past efforts have shown that CDPA is most robust for Y cells (Shapley and Victor, 1978). Our investigation of CDPA in monosynaptically connected Y-type retinal ganglion cells and LGN neurons reveals that LGN neurons exhibit greater CDPA than their retinal inputs. Our results also show that relayed retinal spikes exhibit greater CDPA than non-relayed spikes, suggesting that contrast gain control exerts an influence on spike transmission at the retinogeniculate synapse. To gain insight into the possible mechanisms that underlie the increase in CDPA between the retina and LGN, we developed a model to examine whether temporal filtering of incoming retinal spikes could produce CDPA. In this model, retinal spikes following a short ISI (<30 ms) are more effective in driving a postsynaptic spike than retinal spikes following longer ISIs. Results from this effort revealed that relayed spikes had greater CDPA than non-relayed spikes, although the magnitude of this effect was less than that measured between retinal ganglion cells and LGN neurons *in vivo*. While additional mechanisms, including polysynaptic interactions and local inhibition, likely contribute to increased transience and the increased CDPA of LGN neurons, ISI filtering provides a simple feed-forward mechanism by which gain control can be augmented in the visual pathway.

In summary, simultaneous recordings of monosynaptically connected retinal ganglion cells and LGN neurons showed enhancement of contrast gain control mechanisms by the LGN. Two measures of contrast gain control: (1) increased sensitivity to low-contrast stimuli and (2) CDPA, were both greater for individual LGN neurons compared to their simultaneously recorded retinal inputs. Further analyses suggests that increased sensitivity is achieved via spatial convergence of multiple retinal inputs, while increased CDPA is achieved, in part, on temporal summation of arriving signals. Taken together, these results reveal a breadth of processing strategies, both spatial and temporal, employed by the thalamus to transform visual signals en route from retina to cortex.

## Author Contributions

DLR contributed to experimental design, data collection, data analysis, and manuscript preparation. HJA contributed to data analysis and manuscript preparation. DKW contributed to data analysis and manuscript preparation. WMU contributed to experimental design, data collection and manuscript preparation.

## Funding

This work was supported by National Eye Institute Grants EY 13588, EY12576, and T32 EY15387, and the German ministry for education and research (BMBF) 031 A 308 and 01GQ1002.

## Conflict of Interest Statement

The authors declare that the research was conducted in the absence of any commercial or financial relationships that could be construed as a potential conflict of interest.
